# Comparative histopathologic analysis of COVID-19-associated pseudo-chilblains (“COVID-toes”), with idiopathic pernio, and chilblains lupus erythematosus

**DOI:** 10.1016/j.jdin.2025.03.003

**Published:** 2025-04-21

**Authors:** Taylor Harp, Tionna Szymanski, Haitao Chu, Zheng Wang, Jensen Fisher, Kevin J. Gaddis, David R. Pearson

**Affiliations:** a Departments of Internal Medicine and Dermatology, MedStar Georgetown University- Washington Hospital Center, Washington, District of Columbia; b Rocky Vista University College of Osteopathic Medicine, Parker, Colorado; c Division of Biostatistics, School of Public Health, University of Minnesota, Minneapolis, Minnesota; d Department of Biostatistics and Research Decision Sciences, Merck & Co, Inc, Rahway, New Jersey; e Department of Dermatology, University of Minnesota, Minneapolis, Minnesota

**Keywords:** acral lesions, biopsy, Chilblain-like lesions, chilblains, chilblains lupus, coronavirus, COVID, COVID-19, COVID-associated, COVID-Toes, histopathology, pernio, pernio-like lesions, pseudo-chilblains, severe acute respiratory syndrome coronavirus 2

*To the Editor:* COVID-19-associated pseudo-chilblains (COVID-toes) presents as asymptomatic to tender, violaceous, acral subcutaneous papules. Its association with SARS-CoV-2 is debated because it often affects younger, asymptomatic patients with negative COVID-19 polymerase chain reaction results.^1^ COVID-toes may result from immunologic stimulation, direct viral effect, or an epiphenomenon from self-isolation. This study evaluates histopathology of COVID-toes and compares it to chilblains lupus erythematosus (ChLE) and idiopathic pernio (IP) in order to differentiate them.

A literature search was conducted to identify peer-reviewed, primary observational studies containing histopathologic data for COVID-toes, IP, and ChLE. 50 COVID-toes, 5 IP, and 5 ChLE articles were included. Explicitly reported (present or absent) histopathologic findings from biopsies of clinically diagnosed COVID-toes lesions were compared with IP and ChLE through common effect meta-analysis, using proportions and odds ratios (ORs).

ORs with 95% confidence intervals (CIs) and standardized mean differences (SMD) were calculated for histopathologic findings. Means and ORs with 95% CI were calculated for demographics. Further details Supplemental Materials, available via Mendeley at https://data.mendeley.com/preview/py7n8284gj?a=b0a4b242-c292-4cec-a1b1-9cd443aa3c66.^2^

COVID-toes patients were younger (25.8 years), compared with ChLE (40.76 years) and IP (41.95 years).^2^ Males were 13.2 times more likely to be diagnosed with COVID-toes than females when compared to ChLE (95% CI 5.4, 41.4), and 3.1 times more likely compared to IP (95% CI 1.9, 5.2).^2^

The most common histopathologic findings in COVID-toes were lymphohistiocytic perivascular infiltrate (86.1%), periadnexal infiltrates (61.2%; predominantly perieccrine), apoptotic or necrotic keratinocytes (48.6%), and interface dermatitis (46.9%; predominantly vacuolar). Other common findings were erythrocyte extravasation (45.7%), dermal edema (41.2%), lymphocytic vasculitis (33.1%), reactive endothelia (28.6%), and microthrombi (22%) (Table I and Fig 1).

**Table I:** Aggregated proportions of histopathologic findings of COVID-19-associated pseudo-chilblains, ChLE, and IP

Histopathologic Finding	COVID-toes			ChLE			IP			COVID-toes vs ChLE		COVID-toes vs IP	
	*n*	Total *N*	Proportion (95% CI)	*n*	Total *N*	Proportion (95% CI)	*n*	Total *N*	Proportion (95% CI)	OR∗ (95% CI)	SMD∗	OR∗ (95% CI)	SMD∗
Perivascular infiltrate	211	245	0.861 (0.818, 0.905)	38	62	0.613 (0.492, 0.734)	93	95	0.979 (0.95, 1.008)	**3.901 (2.089, 7.269)**	**0.588**	**0.164 (0.033, 0.504)**	**0.445**
Periadnexal infiltrate	150	245	0.612 (0.551, 0.673)	12	62	0.194 (0.095, 0.292)	55	95	0.579 (0.48, 0.678)	**6.367 (3.352, 12.934)**	**0.944**	1.15 (0.71, 1.855)	0.068
Apoptotic/necrotic keratinocytes	119	245	0.486 (0.423, 0.548)	19	62	0.306 (0.192, 0.421)	46	95	0.484 (0.384, 0.585)	**2.107 (1.183, 3.868)**	0.373	1.006 (0.627, 1.614)	0.003
Interface dermatitis	115	245	0.469 (0.407, 0.532)	40	62	0.645 (0.526, 0.764)	51	95	0.537 (0.437, 0.637)	**0.492 (0.274, 0.865)**	0.36	0.765 (0.475, 1.226)	0.135
Erythrocyte extravasation	112	245	0.457 (0.395, 0.52)	7	62	0.113 (0.034, 0.192)	29	95	0.305 (0.213, 0.398)	**6.236 (2.958, 15.051)**	**0.825**	**1.9 (1.16, 3.167)**	**0.317**
Dermal edema	101	245	0.412 (0.351, 0.474)	14	62	0.226 (0.122, 0.33)	61	95	0.642 (0.546, 0.739)	**2.349 (1.267, 4.588)**	**0.408**	**0.394 (0.24, 0.638)**	**0.473**
Thrombi	54	245	0.22 (0.169, 0.272)	5	62	0.081 (0.013, 0.148)	22	95	0.232 (0.147, 0.316)	**2.975 (1.275, 8.398)**	0.399	0.93 (0.536, 1.65)	0.027
Mucinosis	50	245	0.204 (0.154, 0.255)	8	62	0.129 (0.046, 0.212	35	95	0.368 (0.271, 0.465)	1.656 (0.789, 3.869)	0.202	**0.44 (0.263, 0.74)**	**0.37**
Spongiosis	41	245	0.167 (0.121, 0.214)	3	62	0.048 (0.01, 0.135)	10	95	0.105 (0.044, 0.167	**3.45 (1.267, 12.956)**	0.391	1.652 (0.829, 3.567)	0.182
Reactive endothelia	70	245	0.286 (0.229, 0.342)	NA	NA	NA	1	95	0.011 (0, 0.057)	NA	**NA**	**25.308 (6.743, 225.216)**	**0.84**
Lymphocytic vasculitis	81	245	0.331 (0.272, 0.39)	3	62	0.048 (0.01, 0.135)	2	95	0.021 (0.003, 0.074)	**8.422 (3.171, 31.233)**	**0.772**	**18.529 (6.221, 90.507)**	**0.89**
Vascular ectasia/proliferation	23	245	0.094 (0.057, 0.13)	5	62	0.081 (0.013, 0.148)	16	95	0.168 (0.093, 0.244)	1.104 (0.445, 3.23)	0.047	0.509 (0.259, 1.018)	0.222

COVID-19-associated pseudo-chilblains (COVID-toes). Bold values indicate statistical significance.

∗ Unadjusted odds ratio (OR) between the diagnoses and histopathologic finding and tis 95% confidence interval were estimated by Firth logistic regression to reduce the bias that may by introduced by small numbers. The standardized mean differences (SMDs) for each histopathologic finding between Covid-toes vs CHLE, and between Covid-toes vs IP, were computed based on Yang and Dalton. SMD values >0.8 indicate a large difference between the 2 groups, 0.5-0.8 a medium difference, 0.2-0.5 a small difference, <0.2 a very small difference.

**Fig 1:**
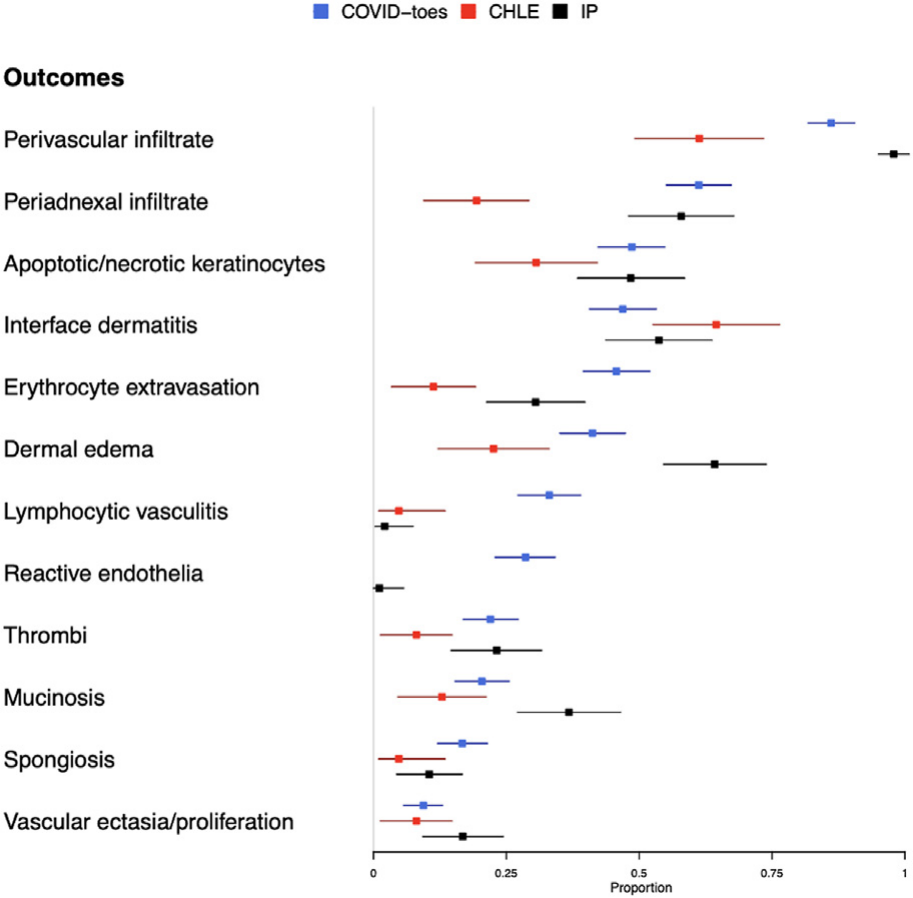
Forest plot of the proportions of histopathologic findings observed in COVID-toes, ChLE, and IP.

Compared to ChLE, COVID-toes patients more often demonstrated perivascular (OR 3.901, 95% CI 2.089-7.269, SMD 0.588) and periadnexal infiltrates (OR 6.367, 95% CI 3.352-12.934, SMD 0.944), but less often had interface dermatitis (OR 0.492, 95% CI 0.274-0.865, SMD 0.36) (Table I and Fig 1). Compared to IP, COVID-toes patients more often demonstrated reactive endothelia (OR 25.308, 95% CI 6.743-225.216, SMD 0.84) and lymphocytic vasculitis (OR 18.529, 95% CI 6.221-90.507, SMD 0.89), but less often showed dermal edema (OR 0.394, 95% CI 0.240-0.638, SMD 0.473) (Table I and Fig 1).

The histopathologic patterns suggest a more vasculocentric inflammatory pattern in COVID-toes, possibly by SARS-CoV-2 infecting endothelium, leading to thrombosis and vasculitis.^3^ It has been postulated that IFN-I is upregulated, causing inflammatory damage, also a proposed mechanism in clustered cases of IP.^2^^,^^4^^,^^5^ Our study does not provide evidence to comment on this. This study is limited by the number of articles available, inconsistent data reporting, and inability for authors to review the slides to confirm findings.

Diagnosis of COVID-toes may be aided by demographics and histopathology. Younger males with vascular damage are more likely to have a diagnosis of COVID-toes, while older patients with perivascular and periadnexal infiltrates, particularly if interface dermatitis is observed, are more likely to have a diagnosis of IP or ChLE.

COVID-toes is a unique condition with distinct histopathology. While more biopsies and research are needed to differentiate these conditions and enhance diagnostic accuracy, this study provides valuable insights for diagnosis and future investigations.

## Conflicts of interest

Dr Pearson is or has been a clinical trial investigator for Bristol-Meyers-Squibb, Corbus Pharmaceuticals, Elorac, Eli Lilly and Company, EMD Serono, Emerald Health Pharmaceuticals, Kadmon, Pfizer, and Soligenix, and has received consulting fees from Biogen and Pfizer within the last 3 years. Dr Wang is employed by a pharmaceutical company, Merck & Co, Inc. Authors Harp, Fisher, Szymanski and Drs Chu and Gaddis have no conflicts of interest to declare.
